# Phytochemicals, Nutrition, Metabolism, Bioavailability, and Health Benefits in Lettuce—A Comprehensive Review

**DOI:** 10.3390/antiox11061158

**Published:** 2022-06-13

**Authors:** Min Shi, Jingyu Gu, Hanjing Wu, Abdur Rauf, Talha Bin Emran, Zidan Khan, Saikat Mitra, Abdullah S. M. Aljohani, Fahad A. Alhumaydhi, Yahya S. Al-Awthan, Omar Bahattab, Muthu Thiruvengadam, Hafiz A. R. Suleria

**Affiliations:** 1 Faculty of Veterinary and Agricultural Sciences, School of Agriculture and Food, The University of Melbourne, Parkville, VIC 3010, Australia; mssh1@student.unimelb.edu.au (M.S.); jingyug@student.unimelb.edu.au (J.G.); hanjingw@student.unimelb.edu.au (H.W.); 2 Department of Chemistry, University of Swabi, Swabi 94640, Pakistan; mashaljcs@yahoo.com; 3 Department of Pharmacy, BGC Trust University Bangladesh, Chittagong 4381, Bangladesh; talhabmb@bgctub.ac.bd; 4 Department of Pharmacy, Faculty of Allied Health Sciences, Daffodil International University, Dhaka 1207, Bangladesh; 5 Department of Pharmacy, International Islamic University Chittagong, Chittagong 4318, Bangladesh; zidankhan9090@gmail.com; 6 Department of Pharmacy, Faculty of Pharmacy, University of Dhaka, Dhaka 1000, Bangladesh; saikatmitradu@gmail.com; 7 Department of Veterinary of Medicine, College of Agriculture and Veterinary Medicine, Qassim University, Buraydah 52571, Saudi Arabia; dr.johani@live.com; 8 Department of Medical Laboratories, College of Applied Medical Science, Qassim University, Buraydah 52571, Saudi Arabia; f.alhumaydhi@qu.edu.sa; 9 Department of Biology, Faculty of Science, University of Tabuk, Tabuk 47512, Saudi Arabia; alawthan@ut.edu.sa (Y.S.A.-A.); obahattab@ut.edu.sa (O.B.); 10 Department of Biology Faculty of Sciences, Ibb University, Ibb 70270, Yemen; 11 Department of Crop Science, College of Sanghuh Life Science, Konkuk University, Seoul 05029, Korea

**Keywords:** *Lactuca sativa* L., bioactive phytochemicals, antioxidant, health benefits, nutrients

## Abstract

Lettuce is one of the most famous leafy vegetables worldwide with lots of applications from food to other specific uses. There are different types in the lettuce group for consumers to choose from. Additionally, lettuce is an excellent source of bioactive compounds such as polyphenols, carotenoids, and chlorophyll with related health benefits. At the same time, nutrient composition and antioxidant compounds are different between lettuce varieties, especially for green and red lettuce types. The benefit of lettuce consumption depends on its composition, particularly antioxidants, which can function as nutrients. The health benefits rely on their biochemical effect when reaching the bloodstream. Some components can be released from the food matrix and altered in the digestive system. Indeed, the bioaccessibility of lettuce is measuring the quantity of these compounds released from the food matrix during digestion, which is important for health-promoting features. Extraction of bioactive compounds is one of the new trends observed in lettuce and is necessarily used for several application fields. Therefore, this review aims to demonstrate the nutritional value of lettuce and its pharmacological properties. Due to their bioaccessibility and bioavailability, the consumer will be able to comprehensively understand choosing a healthier lettuce diet. The common utilization pattern of lettuce extracted nutrients will also be summarized for further direction.

## 1. Introduction

Lettuce (*Lactuca sativa* L.) belongs to the Asteraceae family and originates in the Mediterranean. It is a successful and diverse plant distributed worldwide [1]. The first cultivated lettuce appears in several primitive writings in early 2680 BCE as a medicinal herb. The Asteraceae can be considered the family of plants with a large number of species, around 23,000 to 30,000 [1]. Formally, there are seven different types in the lettuce group, including Cos (a.k.a. Romaine), Butterhead, Leaf (a.k.a. Cutting), Stalk (or Asparagus), Crisphead (a.k.a. Iceberg), Latin, and Oilseed. The whole heads of lettuce and the fresh-cut form are the common products in the market. The colors, shapes, and nutrients are important factors to affect consumer purchase choice [2].

Lettuce is rich in water with 94–95% content and low in calories. It is also an excellent source of vitamins, minerals, and bioactive compounds such as polyphenols, carotenoids, and chlorophyll with related health benefits [3]. Additionally, phytochemical compositions and contents are different in various types. Compared with green lettuce and red lettuce, some studies have reported that red lettuce has comparatively higher phenolic levels than green. Thus, red lettuce is a good source of antioxidants in the daily diet [4]. It was found that lettuce contains a high amount of Vitamin C [5].

Consuming lettuce is beneficial for human health—related to the bioactive compounds in vegetables [6]. Some epidemiological studies have reported that vegetable consumption is associated with a lower risk of chronic diseases, such as cancer and cardiovascular disease [7,8,9,10,11]. Lettuce is an important natural source of phytochemicals. These molecules, which include glycosylated flavonoids, phenolic acids, carotenoids, vitamin B groups, ascorbic acid, tocopherols, and sesquiterpene lactones, are vital bioactive nutritional components [12,13,14,15]. Additionally, the findings demonstrated that the consumption of lettuce preserved in modified-atmosphere packaging did not alter the plasma redox state of healthy participants [16]. Furthermore, several researchers found that hydroponic *Lactuca sativa* can heal diseases such as oxidative damage, cancer, Alzheimer’s disease, and diabetes [17,18,19].

Natural antioxidant compounds extracted and identified from plant sources such as lettuce have become a popular trend because of the general awareness of the importance of a healthy diet and limitations on the use of hazardous synthetic antioxidants [20]. Although, many kinds of research focusing on lettuce, reviews on nutritional highlights, and consolidation of research findings are limited.

Therefore, the objective of this review was to summarize the overview of lettuce, including nutritional compounds, especially bioactive compounds, and potential health benefits, which are essential for consumers to achieve a better daily diet and acquire a better knowledge of the potential health benefits.

## 2. Methodology

**Figure 1:**
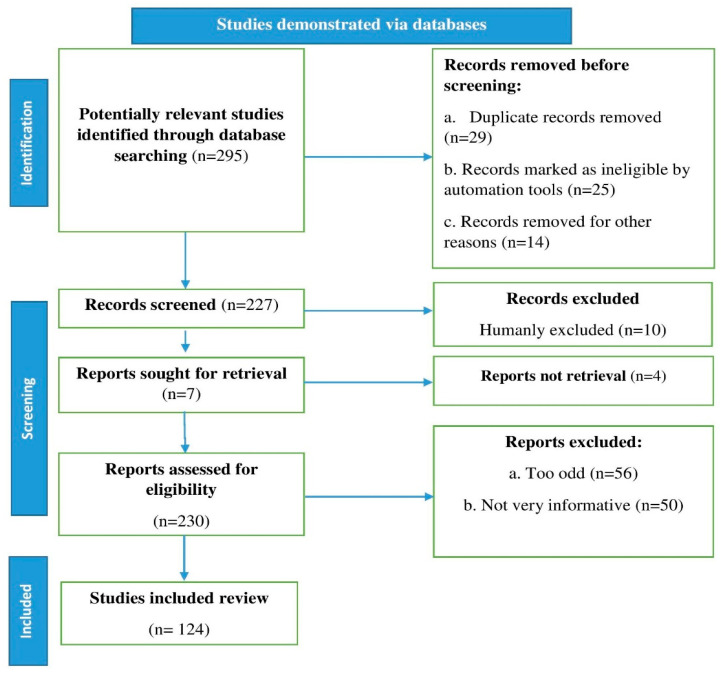
Stages involved in selecting published data for inclusions in the current study are depicted in a flow chart; n: number of literature reports.

We conducted a literature search and found recent relevant references in databases including Scopus, Science Direct, Elsevier, PubMed, and Web of Science. Our search utilized the terms lettuce, phytochemicals, nutrition, metabolism, health benefits, and bioavailability. Research reports, review articles, and original research articles in English were selected and evaluated. We also looked over the citations and inserted them where appropriate. Page et al. [21] recommended using an algorithm that inserted all of the processes involved in identifying the relevant material in the study, as shown in the flow chart in Figure 1.

## 3. Lettuce Types and Production

**Figure 2:**
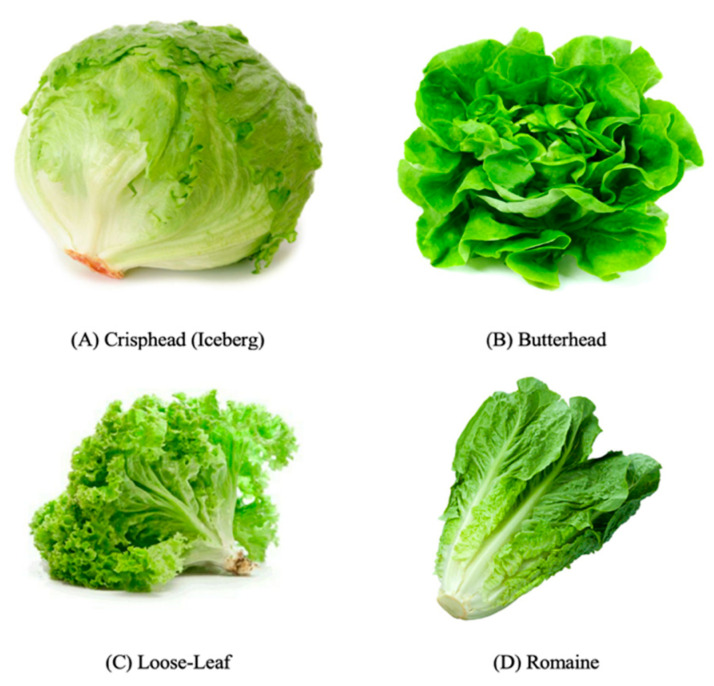
The frequent growth types of lettuce: Crisphead, Butterhead, Looseleaf, and Romaine.

In Australia, Crisphead (iceberg), romaine (cos), butterhead, and looseleaf are the most common groups, while there are significant differences in growth habit, leaf color, shape, and texture between them (Figure 2).

Countries such as America, Asia, Australia, and Europe have become more vigorous markets for lettuce [22]. The gross yield of lettuce in China is the maximum globally, occupying 56.4% of the harvesting market, followed by the United States (14.3%) and India (4.1%) [23]. In Australia, the production and consumption of lettuce have increased. Lettuce production increased by about 118% between 1980 and 2005, while other vegetables such as maize, potato, paddy rice, and a tomato increased by 149%, 135%, 146%, and 128%, respectively [24]. Compared to yield increases, the increased production was mainly attributed to an increasing area cultivated and improving the resistance to insects and disease. About 6000 hectares are cultivated annually in Australia, manufacturing 135,000 tonnes for different markets, including fresh and processing. It is estimated that the lettuce industry in Australia is worth around $100 million and $8 million belongs to export.

## 4. Taxonomical Classification

Kingdom—Plantae

Subkingdom—Viridiplantae

Infrakingdom—Streptophyta

Superdivision—Embryophyta

Division—Tracheophyta

Subdivision—Spermatophytina

Class—Magnoliopsida

Superorder—Asteranae

Order—Asterales

Family—Asteraceae

Genus—*Lactuca* L.

Species—Lactuca sativa L.

### 4.1. Crisphead

It is also known as the iceberg, one species of *Lactuca sativa* var. *capitata* family, which forms a tight and dense head of spherical leaves that fold each other. It is known for its thick and crisp leaves and prominent veins. The wide range of leaf colors from light green to deep green and most varieties are mid-green during the mature period, while some specific genotypes contain anthocyanins [25].

### 4.2. Butterhead

Butterhead also belongs to *Lactuca sativa* var. *capitata* and shows a small head of spherical leaves, such as Crisphead, which the butterhead group can also form, but the leaves are not folded tightly. The inner leaves are cream green due to the lack of light, while the outer leaves are darker colored or brownish and easier to be torn and bruised than the Crisphead group. Their pliability and oily leaf texture are attractive to the consumer.

### 4.3. Loose-Leaf

It is also known as a leaf (*Lactuca sativa*). These groups form no heads but have many leaves in the center of lettuce that include smooth margins, deeply lobed, or even curled and fringed leaves. The leaf lettuce is characterized by shorter and softer leaves than romaine that are more perishable during the shipment. Looseleaf lettuce can be marketed as a whole product and salad mix ingredient, usually classified into three types: green, red, and oak. The various leaves’ colors range from green to yellowish-green and some contain a red shade relying on the content of anthocyanins in lettuce and the light intensity used when growth [24,26].

### 4.4. Romaine

Romaine, also called Cos or *Lactuca sativa* L., is the most upright growing one of the four major types of lettuce. This specie forms an oblong-shaped head with long and rigid leaves with a prominent midrib, which is upright, and can grow to a maximum of 30 cm [27]. The outer leaves are medium green, while the leaves folded tightly are greenish-white in the center. Their sweet flavor is more apparent than in any other lettuce cultivar (Figure 1). Many countries worldwide cultivate lettuce that can be classified into several consumer uses such as whole head, bulk harvest (for salad manufacturing or food service), and baby leaf [28].

## 5. Phytochemicals and Nutritional Composition of Lettuce

### 5.1. Phenolic Compounds

Phenolic compounds are vital to plant secondary metabolites, which play an important role in the plant protection mechanism [29]. It is believed that phenolics can prolong the lettuce shelf life and improve plant stress resistance, contributing to decreasing the loss of postharvest [30]. This is characteristic because the reactive oxygen species can be scavenged, which is believed to be involved in leaf senescence and also in the plant’s antioxidant defense mechanism [31]. Firstly, the most common phenolic acids in lettuce are caffeic acid, chlorogenic acid, as well as their derivatives [32]. Phenolic acids account for 70% and 94% of the total phenolic content in some green varieties, whereas only 35% and 45% for red lettuce [33]. Secondly, flavonoid composition and flavonols in lettuce are quercetin and kaempferol derivatives, anthocyanins, and flavone luteolin [33]. Furthermore, quercetin (flavonol) was followed by isorhamnetin, an essential (*O*-methylated flavonol) flavonoid in some lettuce types. There are free and bound phenolic compounds found in lettuce [31]. The free phenolic compounds that can be identified in the methanolic extract were protocatechuic, chlorogenic, caffeic and p-coumaric acids, kaempferol, luteolin, apigenin, and phlorizin. Chlorogenic acid is the composition mainly found among the free phenolics. Generally, containing the highest chlorogenic and caffeic acids were romaine lettuces, but with great variability among cultivars [31].

Another main difference between red and green lettuce is anthocyanins since this type of flavonoids is responsible for the red/blue/purple color pigments in some vegetables and fruits, but not all [2]. There was an association between the color of leaves and lettuce type. Generally, lettuce’s qualitative and quantitative phenolic content relies on their variety with different genetic information. In recent years, red lettuce has been increasingly popular in the diet as a salad mix ingredient because of its anthocyanin content and higher phenolic contents that contribute to greater health benefits than the green one [34]. However, various agronomic or environmental factors such as light, and climatic and postharvest conditions and their tissue type can influence the phenolic content found in lettuce (Table 1) [35]. Therefore, further studies can focus on this area. Recent research has shown that a number of dietary polyphenolic compounds produced from plants are more potent antioxidants in vitro than vitamins E or C and may therefore contribute to the protective benefits in vivo. It is now feasible to establish the antioxidant activities of plant-derived flavonoids in the aqueous and lipophilic phases and to determine the degree to which the overall antioxidant potentials of wine and tea may be explained by the activities of individual polyphenols [36].

### 5.2. Carotenoids

Carotenoids are a kind of lipid-soluble pigment widely found in fruits and vegetables that are yellow-orange and leafy vegetables with dark color [45]. β-carotene content was highest presented in all the lettuce cultivars, accounting for half of the total carotenoids, which was followed by lutein (20%), lactucaxanthin (13%), violaxanthin (11%), and neoxanthin (6%) [31]. However, the content of carotenoids is also varying in different lettuce types. There was a moderate association between β-carotene content and leaf color value L*, suggesting that the increasing leaf brightness or luminosity is highly related to β-carotene content [2]. Carnat et al. [46] reported that the amount of β-carotene and lutein were generally higher in red oak leaf than in green lettuce types such as butterhead and Batavia, which confirmed that the carotenoid concentration and the green color chlorophyll content are positively correlated [47]. In contrast, these findings remain controversial in other academic studies. For example, Mou [48] indicated higher levels of carotenoids in green leaf lettuce than the red lettuce, and a similar content of β-carotene is found in red leaf lettuce and the green one. Therefore, the carotenoid contents are not completely related to leaf pigmentation.

In addition, previous reports have demonstrated that cultivar and other conditions could affect the carotenoid content of lettuce. As for Crisphead with a closed head shape, its leaves obtain less light than the leaves with a relatively open head, this leads to less carotenoid synthesis [48]. The outer leaves’ carotenoids are higher than the inner leaves because of the positive influences of light intensity on carotenoid biosynthesis in the outer leaves [49].

### 5.3. Chlorophyll

As the main pigment of leafy green vegetables, chlorophyll plays a significant role in determining the health of vegetables. The chlorophyll content quantification can be considered a visible factor in monitoring the nutritional statement of green leafy vegetables such as lettuce since most of the nitrogen is integrated into leaf chlorophyll. In addition, with the senescence after harvest, lettuce chlorophyll will gradually be degraded, which can be considered an indicator of quality [35]. Moreover, chlorophyll is thought to be a good source of vitamins E, A, C, K, and β-carotene, and important minerals such as magnesium, potassium, iron, calcium, and essential fatty acids. Chlorophyll has been claimed to have protective effects such as anti-carcinogenic and antimutagenic activities because of its antioxidant properties [50].

López et al. [31] demonstrated the differences found in some types of nutritional and bioactive compounds due to the various structures and sizes of the lettuce head. For example, open lettuce heads, such as Romaine with a higher photosynthetic area, had increased compounds such as chlorophylls, sugars, and other relevant metabolites [51]. In contrast, the cultivars having closed heads can block the sunlight penetration, resulting in a lower content of metabolites. On the basis of Mou [48], similar effects of head structure can be observed on carotenoid content, which has been mentioned before. The relationship between β-carotene and chlorophyll plays a part in photosynthesis, while the former acts as an accessory pigment. β-carotene can absorb light energy from the chlorophyll at various wavelengths and transfer it to the chlorophyll [52]. Chlorophyll is the main photosynthetic pigment that produces the green color in plants and many algae. Thus, a higher total chlorophyll content can be found in green lettuce varieties than in red ones.

### 5.4. Vitamins

Vitamins are one of the important micronutrients needed for metabolism [53]. In lettuce, folate, and vitamins C and E are the most common types. Firstly, Wang et al. [54] reported total folate is higher in romaine lettuce than in spinach or another popular leafy vegetable. Secondly, two forms of vitamin C, ascorbic acid (AA) and dehydroascorbic (DHAA), both show biological functions in lettuce [9,55]. The simultaneous analysis of both formations is necessary. For instance, ascorbic acid also contributes to 24.5% of the overall antioxidant activity in lettuce, which shows that vitamin C is an essential antioxidant [46]. In contrast, Szeto et al. [56] found that the level of vitamin C in lettuce accounts for only 1% of the total antioxidant power. Such distinct findings can be clarified by having dehydroascorbic acid in lettuce. Even though dehydroascorbic acid seems like vitamin C, it has no antioxidant activity [46]. Unlike folate, lettuce is not a rich source of vitamin C like other vegetables such as broccoli, cauliflower, or pepper [57]. Thirdly, vitamin E is found in different forms of tocopherols among four lettuce [58]. Loose-leaf has the highest content of 1.06 mg/100 g of edible weight. Indeed, α-tocopherols and γ-tocopherols are the principal forms in lettuce and the former is the most biologically active. In addition, Szymańska and Kruk [59] indicated that α-tocopherol dominates in lettuce leaves, while γ-isomer is abundant in part of the lettuce head. Based on the United States Department of Agriculture (New York), the total tocopherol in lettuce is about 0.5 μg/g^−1^ with 0.26 between α- and γ-conformation [60]. In addition, Saini et al. [61] observed that the high content of ascorbic acid and tocopherol in baby-leaf lettuce contributes to the shelf life, preserving phenolic compounds.

### 5.5. Minerals

In terms of minerals, it is the best way to eat many leafy vegetables in the diet to obtain abundant minerals in the body [62]. It is believed that sodium (Na) and potassium (K) play a crucial role in the balance of water and electrolytes and other metabolic functions [63]. Using the recommended consumption of Na each day as the standard, the content of Na from lettuce is low [64]. In contrast, the content of K in lettuce is higher than that of Na in lettuce. In addition, calcium (Ca) is another important mineral that benefits bone health and minimizes the risk of osteoporosis. Other minerals such as phosphorus (P), magnesium (Mg), iron (Fe), and zinc (Zn) can also be found in lettuce and vary from the head type and are affected by soil conditions. Similarly, Santos et al. [65] also found that the primary mineral in lettuce is iron (Fe). Meanwhile, the content of potassium (K) and calcium (Ca) are also the highest, but low in Na.

### 5.6. Anti-Nutrients Compounds

Anti-nutrients are also detected in lettuce species. Such compounds have direct and indirect effects ranging from a mild reaction to a death, which need to be considered. The main anti-nutrients in lettuce include nitrates, phytates, tannins, oxalates, and cyanogenic glycosides. For instance, nitrogen is important for the nutrition and function of lettuce so that plants can influence the metabolic control of lettuce by nitrate and other forms of nitrogen. It is principally detected in cell vacuoles and can be transported in the xylem [66]. The water and nutrients can be carried by xylem from roots to leaves, while the photosynthetic product can be carried by phloem between the leaves and the growth points of the plant, affecting the distribution of nitrate, which means the leaf crops such as lettuce and spinach have relatively high nitrate concentrations. Another influence of this transport mechanism is that old leaves have higher nitrate concentrations than younger leaves [67]. The accumulation of nitrate can cause harmful actions. Another anti-nutrient in lettuce is alkaloids, which are the natural bitter compositions of plants with some pharmacological properties. Alkaloids result in gastrointestinal tract and nervous system disorders that disrupt or improperly augment electrochemical transmission [68]. It is evident that alkaloids have a physiological impact on humans [69]. For example, high tropane alkaloids intake can result in heartbeat rapidly, paralysis, and even death in fatal cases. In addition, a high dose of tryptamines alkaloids can cause staggering and death.

Many plants, including some crops, contain plant toxins that are natural compounds. In many cases, these toxins can be considered defense mechanisms that plants produce to prevent insects from overeating. If one of the plants were eaten enough by an insect, the toxic chemicals could accumulate in their body and reach a high level causing the disrupted function of cells and tissue, then the insect will die. In this way, plant toxins are vital to the natural balance system [70]. In addition, green leafy vegetables containing anti-nutrients such as nitrates, oxalates, phytates, cyanogenic glycosides, and tannins influence micronutrient absorption. Some thermal processing can decrease the content of anti-nutrients in lettuce by boiling, cooking, and blanching. Further research is also necessary on various varieties and adopting agronomic practices that can decrease the content and impact of anti-nutrients in green leafy vegetables such as lettuce and improve their nutritional value.

## 6. Bioavailability of Bioactive Compounds from Lettuce

It is critical to mention the ultimate delivery of these compounds when considering food rich in bioactive compounds and their related health, and thus bioavailability is introduced as a parameter. The definition of bioavailability is the extent to which ingested bioactive compounds reach the circulatory system and act on specific sites [71]. Furthermore, bioaccessibility means the process by which the fraction of bioactive compounds are released from the food matrix through the upper digestive system [72]. Chlorogenic acid, which is the ester of caffeic acid and quinic acid, is one of the most prevalent polyphenols in the human diet, being found mostly in coffee, fruits, and vegetables [72]. For eight days, four groups of rats were given either a food enriched with chlorogenic, caffeic, or quinic acids (250 μmol/d) or a control diet. HPLC–electrospray ionization–tandem mass spectrometry was used to determine the parent chemicals and their metabolites in urine and plasma. There were significant disparities in their levels across the groups.

The recovery of chlorogenic acid in urine was minimal and the total urinary excretion of caffeic acid freed by chlorogenic acid hydrolysis and its tissular methylated metabolites (ferulic and isoferulic acids) was less than 0.5% (mol/mol) of the amount consumed. On the other hand, m-coumaric acid and derivatives of phenylpropionic, benzoic, and hippuric acids were the most abundant metabolites in both urine and plasma. Hippuric acid is derived primarily from the quinic acid moiety, whereas all other metabolites are derived from the caffeic acid moiety. These microbial metabolites account for 57.4% of chlorogenic acid consumption (mol/mol). Such a significant quantity of microbial metabolites indicates that chlorogenic acid’s bioavailability is highly dependent on its metabolism by the gut microflora. They are highlighted for their possible role in understanding the biological effects of dietary polyphenols [73]. Caffeic acid has a 14.7% absolute bioavailability in rats and a 12.4% intestinal absorption in the caco-2 cell model [74]. However, other research discovered that caffeic acid, p-coumaric acid, and ferulic acid are much more bioavailable than chlorogenic acid, despite being excreted faster [75]. The bioavailability of ferulic acid in humans was investigated by tracking the pharmacokinetics of excretion in proportion to intake. The findings indicate that the peak period for maximum urinary excretion is around 7 h and that the recovery of ferulic acid in the urine is approximately 11–25% of that consumed, based on the total free ferulic acid and feruloyl glucuronide excreted [76]. According to a literature review on kaempferol digestion and absorption, the most absorbable forms include kaempferol rutinoside and glucoside in tea, endive glucuronide and glucoside, and broccoli sophoroside. Kaempferol metabolites were detected in the plasma as aglycone and glucuronide, and in the urine as sulfate; however, further human investigations are required to measure kaempferol absorption from meals [77]. Apigenin obtained from food enters the human circulation in vivo and may therefore display physiologic effects [78].

## 7. Digestion, Absorption, and Metabolism

**Figure 3:**
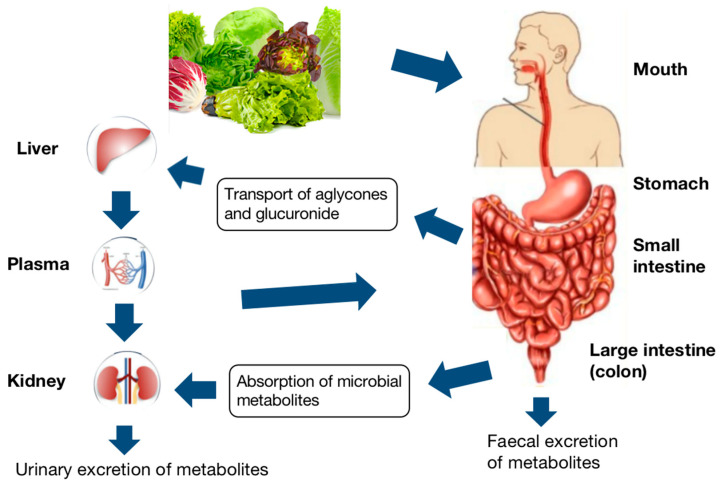
Schematic layout of the digestive pathway of phenolic compounds [81].

Bioactive compounds absorption process from food starts with the chewing and digestion of food in the mouth, which is the oral phase. The roles of chewing and digestive enzymes, especially α-amylase, play in reducing food particle size and the beginning of phenolic compounds release [79]. After that, the digest will transfer to the stomach, where the gastric phase begins. During this phase, the pepsin, as the chief digestive enzyme in the stomach, acidic pH, and mixing can enhance the breakdown of the food matrix, causing further release [80]. Following the gastric phase, the gastric chyme is gradually released into the upper small intestine, also called the duodenum, where the small intestine phase begins under an alkaline environment. At this phase, the pancreas and liver release enzymes and bile salts enhance the most food degradation. As the food matrix is completely digested into absorbable macronutrient units, further compounds will subsequently be released. The undigested phenolic compounds continue to move to the lower intestinal compartments, which are the colon parts, where they can further metabolize by native microbiota. The produced metabolites can circulate in the bloodstream or be delivered to target tissues by plasma proteins and lipoproteins. The metabolism of phenolic compounds in humans, as an example, was summarized in Figure 3.

### 7.1. Phenolic Compounds

In nature, phenolics are mainly present in the form of esters, glycosides, and polymers, which need to be hydrolyzed and metabolized in the environment or by the natural microbiota of the digestive system before being absorbed [81]. Their bioavailability regulates the release and activation of phenolic compounds, and the metabolic form is transferred to the target organ through the blood and then passes through the gastrointestinal tract. According to previous literature, phenolic compounds are digested around 48% in the stomach and intestinal stages, with the remaining 42% bioaccessible in the colon [82]. They also reported that 10% of phenolic compounds are undigested and remain intact within the food matrix. As for most phenolic compounds, only the aglycone form with highly hydrophobic characteristics can pass across physiological membranes through diffusion. In addition, the glycosidic form of phenolic compounds has undoubtedly influenced absorption in the intestine phase [83].

### 7.2. Carotenoids

Carotenoids will be released from the food matrix and then incorporated into the mixed micelles that are formed by carotenoids, acylglycerols, cholesterol, and phospholipids before they can be absorbed. The mixed micelles can transport liposoluble compounds to the intestinal epithelium [84]. Hence, the formation of micelles can be considered one of the important parameters that influence the bioavailability of carotenoids. Furthermore, Hof et al. [85] demonstrated that the bioaccessibility of carotenoids in animals and humans is quite low due to the structure of carotenoids providing interaction with other compounds in the food matrix.

### 7.3. Vitamin C

There are reduced and oxidized forms in vitamin C. Both can be digested and absorbed throughout the small intestinal epithelium by various mechanisms. A sodium-dependent active transporter (SVCT1) was used for ascorbic acid absorption [86]. Additionally, with the help of a glucose transporter, dehydroascorbic acid can be absorbed by promoting diffusion in the duodenum and jejunum [87].

### 7.4. Factors Affecting the Bioavailability

**Figure 4:**
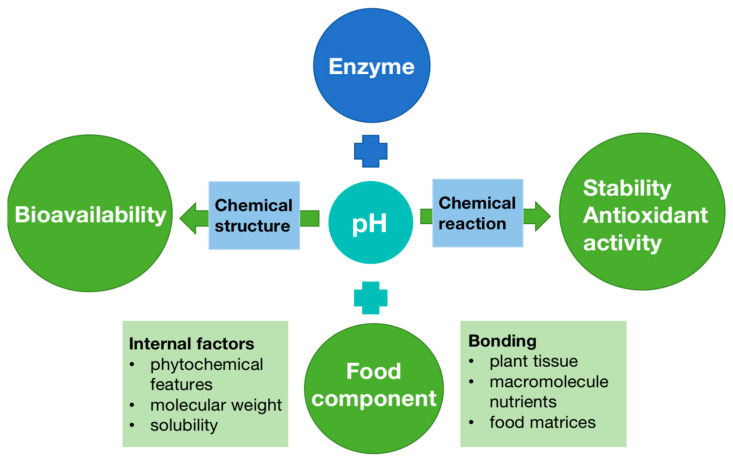
Parameters affecting the bioavailability of bioactive compounds [89].

The internal and external parameters can potentially affect the ultimate biological activity of bioactive compounds in the diet (Figure 3). For example, enzyme action is another important factor, which causes molecular transformation in compounds depending on their solubility as well as destroying chemical bonds in phenolics and proteins, carbohydrates, and lipids with cell wall tissues, thereby enhancing the phenolic compounds released [88]. A different pH in each phase environment may impact a fraction of the bioactive compounds by degradation, modification, or transformation of compounds into various structural forms with distinct chemical characteristics, bioaccessibility, bioavailability, and biological activities. Moreover, when food comes into the mouth and ingestion starts, different compounds release from the food matrix, with interactions that influence phytochemical bioavailability (Figure 4).

## 8. Health Benefits and Risks of Lettuce

Patients with severe or end-stage renal illness exhibit uremic pruritus often and prominently. The lack of effective therapeutics for pruritus associated with a renal illness often causes several complications for these individuals and makes it difficult to choose a suitable treatment. Using a molecular docking approach, researchers explored the interactions between nineteen natural bioactive components of lettuce (*Lactuca sativa* L.) and human kappa opioid receptors. According to the in silico docking experiments, most ligands conferred greater antipruritic effectiveness than gabapentin. γ-tocopherol, δ-tocopherol, and campesterol had the greatest protein-binding affinities [90].

**Figure 5:**
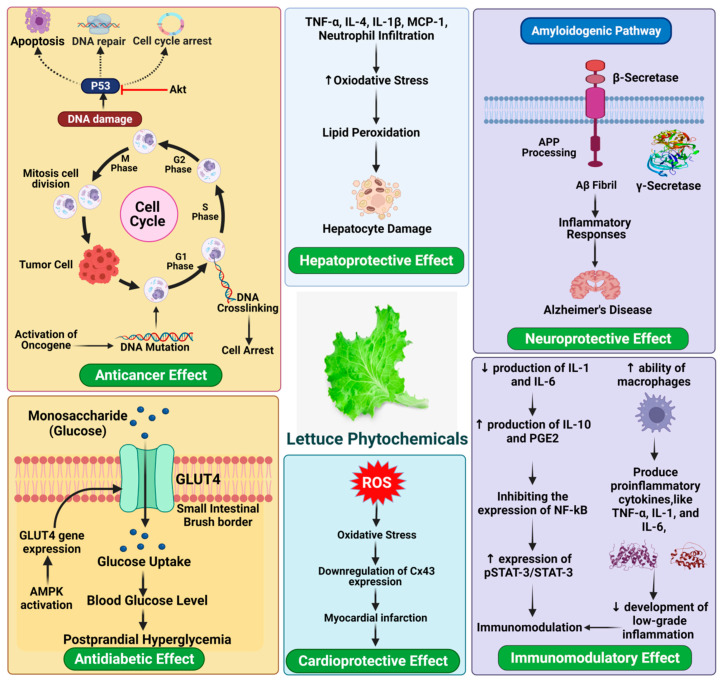
Health-promoting benefits of lettuce.

Various kinds of lettuces on the market provide texture, taste, and flavor and provide a variety of phytochemicals with related health benefits [91]. The frequency of consumption means that lettuce nutrients account for a considerable proportion of dietary intake. Altunkaya et al. [92] observed a synergistic antioxidant effect in lettuce, which via inhibiting polyphenol oxidase (PPO) activity associated with quercetin and α-tocopherol. Meanwhile, the existence of anthocyanins in iceberg lettuce triggers the antioxidant capacity, through both enzyme inhibition and the sequestration of trace elements involved in the production of free radicals [93,94]. Consequently, the antioxidative combination of several bioactive compounds in lettuce contributes to numerous pharmacological properties, including cardio-protective effect, anti-cancer, and anti-diabetic (Figure 5).

### 8.1. Healthy Benefits

#### 8.1.1. Cardio-Protective Effect

Cardiovascular diseases (CVDs) are types of chronic non-infectious diseases that are one of the major threats to people’s health, and are related to a large number of complex risk factors such as hypertension, hyperlipidemia, diabetes, and metabolic syndrome [95]. Some previous epidemiological studies prove that consuming vegetables is positively related to treating CVDs, especially for some green leafy vegetables such as lettuce. For example, Nicolle et al. [96] investigated the diet of rats containing 20% lettuce can have a cardio-protective effect by improving cholesterol metabolism and plasma antioxidant ability.

Lettuce tends to have a number of interesting impacts on CVD factors in various mechanisms owing to its fiber, the availability of antioxidants, and probably other phytochemicals. It is believed that fiber and antioxidant compounds have positive effects on cholesterol metabolism and the prevention of CVDs. Firstly, the lower cholesterol impact of lettuce can be attributed to the fiber part. Pectin, soluble fibers affecting lipid metabolism both in animal and human bodies, has been verified to reduce digestive cholesterol absorption [97]. The cholesterol absorption inhibition mechanisms primarily involve disrupting micelle formation and delaying the cholesterol transfer through the unstirred layer [98]. Hypocholesterolemia impacts can also be found in dietary fiber by promoting fecal excretion of overall steroids as bile acids. That reflects the retention of bile acids in the viscous medium and the acceleration of biliary flow. In addition, there are also some indirect impacts on the cholesterol mechanism exerted by fiber. Short-chain fatty acids such as propionate, which is considered the most effective cholesterol-lowering agent, are produced by fiber fermentation in the large intestine. All these mechanisms combined can reduce the accumulation of plasma cholesterol [96]. Secondly, lettuce can also provide many antioxidant compounds, such as vitamin E, C, carotenoids, and polyphenols, which have health benefits [99]. The heart’s defense against lipid peroxidation can have protective effects against cardiovascular diseases, while the heart is one of the vital target tissues for reactive oxygen species (ROS). Nicolle et al. [96] measured that lower peroxidation in the heart has a relationship with the increase in the vitamin E/TG ratio in the plasma. After consuming lettuce, the strong antioxidant ability in plasma was related to a significant increase in vitamin C and vitamin E, and is less effective than vitamin C in the post-prandial period in improving the antioxidant ability. However, various phenolic compounds in lettuce may also improve the antioxidant capacity. LDL oxidation, which can be inhibited effectively by polyphenols in lettuce, is one of the main causes of atherosclerosis [100]. García-Lafuente et al. [101] reported that other mechanisms of phenols to protect against CVDs are antiplatelet, anti-inflammatory effects, increasing HDL, and improving endothelial function.

#### 8.1.2. Anti-Cancer Effect

Lettuce can positively affect cancer prevention because it contains some phytochemicals, and some clinic researchers have stated that these have health benefits. Iodine-biofortified lettuce causes cancer cells to produce reactive oxygen species (ROS), which leads to anticancer effects via the activation of programmed cell death in cancer cells [102].

Firstly, β-carotene and ascorbic acid are potential nutrients in lettuce that can decline the risk of colorectal cancer. Case-control research has been conducted in Italy, having 1584 colorectal patients with a family history of disease and 2879 control objectives. Assessing the daily food consumption suggested an adverse relationship between lettuce consumption and colorectal cancer. Whereas Ca, vitamin E, and folate in lettuce have no significant association with this cancer [103]. Moreover, an investigation of non-smoking patients reported that a daily diet with lettuce could reduce lung cancer incidence owing to carotenoids, β-carotene, and retinol [104]. Secondly, the chlorophyll pigment in green varieties with dark colors can have a protective effect against specific cancer, such as colon cancer and liver cancers. The mechanism of chlorophyll is binding together hydrocarbons, aflatoxins, and other hydrophobic molecules, which are related to cancer, rather than eliminating them [105]. In contrast, other studies have claimed that low consumption of green leafy vegetables in the diet could increase cancer risk. Thirdly, lettuce contains a high content of phenolic acids, dicaffeoyl tartaric acid, and chlorogenic acid, while having a low content of flavonols, namely, quercetin 3-*O*-glucuronide [106]. One of the most vital utilizations of plant-derived therapeutics formed by phenols is for preventing cancer and chemotherapy. Polyphenols can decline the growth rate of tumors, thus having an anti-cancer effect [107,108,109]. The anti-cancer mechanism has been reported by Terry [110], whereby chemoprevention impacts polyphenols such as antioxidants, reducing free radicals and ROS, thus decreasing their damaging effects on DNA. The MTT (3-(4,5-dimethylthiazol-2-yl)-2,5-diphenyltetrazolium bromide) test and flow cytometry were used to determine the antiproliferative and apoptotic effects while simultaneously studying the genes governing apoptosis and cell cycle in this cell culture model. Additionally, we examined the inhibitory and kinetic properties of the aqueous *L. taraxacifolia* (African lettuce) extract using recombinant human CYP450 isozyme model systems (CYP1A2, CYP2C9, and CYP2C19). *L. taraxacifolia* significantly inhibited the development of WHC01 cancer cells. The majority of genes involved in the cell cycle were downregulated. A cell cycle study revealed that *L. taraxacifolia* extract induced a G0-G1 cell cycle arrest in WHC01 cells, followed by morphological alterations. The treatment with *L. taraxacifolia* extract resulted in a 50–70% reduction in the expression levels of CYP1A2 and CYP2C19. In a reversible and time-dependent manner, *L. taraxacifolia* extract inhibited recombinant *CYP1A2*, *CYP2C9*, and *CYP2C19*. This research sheds fresh light on the potential anticancer properties of *L. taraxacifolia*, an extensively utilized medicinal plant in regions of Africa and around the globe, particularly by cancer patients [111]. The anti-proliferative effects of phenolic extracts from lettuce grown under low nitrogen conditions were superior to those of phenolic extracts from lettuce grown under adequate nitrogen conditions, in part because they interfered with the cell cycle and induced apoptosis, when compared to those of phenolic extracts from lettuce grown under adequate nitrogen conditions [112]. Apigenin is a flavonoid produced from edible plants that has been shown to be effective as an anticancer drug in a number of experimental and biological investigations. In many kinds of tumors, including breast, lung, liver, skin, and blood cancers, as well as prostate cancer and pancreatic cancer and cervical cancer and stomach cancer, it causes cell development to be arrested and apoptosis to occur via altering multiple signaling pathways. Apigenin promotes apoptosis by the activation of the extrinsic caspase-dependent pathway, which is characterized by the upregulation of the mRNA expressions of caspase-3, caspase-8, and TNF-α. It stimulates the intrinsic apoptotic pathway, as indicated by the elevation of cytochrome c, Bax, and caspase-3 in human prostate cancer PC-3 cells, but the levels of caspase-8, TNF-α, and B-cell lymphoma 2 remained unaltered. Apigenin therapy results in a considerable downregulation of matrix metallopeptidases-2, -9, Snail, and Slug, which inhibits the invasion of cancer cells. After treatment with apigenin, the expression of the transcription factors NF-κB p105/p50, PI3K, and Akt and the phosphorylation of p-Akt all drop. On the other hand, Apigenin-mediated therapy drastically lowers the expression of the pluripotency marker Oct3/4 protein, which may be related to the downregulation of PI3K/Akt/NF-κB signaling in the cells [113].

#### 8.1.3. Anti-Diabetic Effect

One of the treatment plans can target diabetes, such as α-amylase and α-glucosidase [114,115], in which starch can be hydrolyzed into glucose entering the blood circulation. Thereby, some interesting studies investigated the natural bioactive compounds having an anti-diabetic effect. Lettuce leaves can be considered a phytonutrient storehouse with high carotenoid content such as lutein, lactucaxanthin, and β-carotene [33], while lactucaxanthin is not commonly found in other plants. Gopal et al. [116] researched that the purity of lactucaxanthin separated from lettuce is 96% verified by HPLC and LCMS. This molecule can inhibit α-amylase and α-glucosidase activities, providing a way to reduce post-prandial hyperglycemia by controlling the breakdown of starch during digestion. On the other hand, plants rich in polyphenols also have the effect of anti-diabetic. The inhibitory effect of polyphenols on intestinal glycosidases and glucose transporter has been researched by Dembinska-Kiec, et al. [117]. Cheng et al. [118] experimented that some obese hyperglycemic mice fed with the diet with the addition of red lettuce varieties at the dose of 100 or 300 mg/kg to alleviate diabetes diseases due to the high content of polyphenols. Anthocyanins, as the polyphenolic pigments, were attributed to the red color of lettuce with antioxidant, anti-inflammatory, and anti-diabetes effects. Dietary addition anthocyanins extracted from bilberry and lettuce can promote hyperglycemia and insulin sensitivity in mice with type 2 diabetes. Furthermore, dietary supplementation of anthocyanin cyanidin 3-glucoside feeding for 8 weeks by diabetic mice can decrease the damage of hepatic oxidants and avoid hepatic steatosis [119]. A similar result was found in another study, the addition of anthocyanin 3-glucoside to the high-fat diet feeding for 5 weeks of mice can decrease the level of blood glucose, increase insulin sensitivity, attenuate liver steatosis, and reduces inflammatory cytokines in adipose tissue [120].

#### 8.1.4. Immunomodulatory Effect

In one experiment, the polysaccharides from stem lettuce (SLP) were further purified by anion exchange chromatography followed by size exclusion chromatography to provide two pure polysaccharides, SLP-1 and SLP-2. Galacturonic acid, galactose, and arabinose were the most abundant sugars in SLP-1, which had a molecular weight of 90 KDa and a molar ratio of 17.6:41.7:33.9. In the molar ratio of 11.5:69.5:9.3:8.2, mannose, galacturonic acid, galactose, and arabinose were the primary constituents of SLP-2, which had a molecular weight of 44 KDa and was mostly consisted of mannose, galacturonic acid, galactose, and arabinose. Furthermore, both pure polysaccharides include sulfate radicals, have triple-helical topologies, and have the ability to enhance macrophage proliferation without causing cytotoxicity in the presence of other factors. SLP-2 was shown to be more effective than SLP-1 in stimulating phagocytic and nitric oxide production. As a consequence of the findings, it is possible that polysaccharides from stem lettuce may be investigated as immunomodulatory agents in the pharmaceutical and functional food industries [121]. A study on the murine macrophage cell line RAW264.7 was conducted to determine the immunomodulatory properties of Green lettuce extracts (GLE) and their probable mechanisms of action. GLE considerably boosted NO levels in RAW264.7 cells and the expression of immunomodulators such as iNOS, COX-2, IL-1, IL-6, IL-12, TNF-alpha, and MCP-1 in the cells. GLE was also shown to increase NO levels in RAW264.7 cells dramatically. Despite the fact that GLE stimulated ERK1/2, p38, JNK, and NF-κB, GLE-mediated production of immunomodulators was reliant on p38, JNK, and NF-κB for its effectiveness. In addition, TLR4 suppression prevented the GLE-mediated production of immunomodulators as well as activation of the p38, JNK, and NF-κB transcriptional factors. Together, our findings revealed that the TLR4-MAPK/NF-κB-signaling pathways were involved in GLE-induced macrophage activation and that GLE might be developed as a potential immunomodulatory functional food ingredient [122,123].

#### 8.1.5. Hepatoprotective Effect

The preventive effect of *L. serriola* against paracetamol-induced hepatotoxicity has been shown. Silymarin was prescribed as a routine medication. The hepatoprotective impact of the extract was assessed using biochemical markers in the liver, antioxidant enzymes, and a lipid profile in the blood. It was discovered that the methanolic extract was the most powerful in in vitro antioxidant experiments. Treatment with paracetamol raised the levels of hepatic biomarkers and the lipid profile of the blood while decreasing the levels of antioxidant enzymes. Restoration of liver biomarkers, blood lipid profile, and antioxidant enzymes was seen after pre-treatment with *Lactuca serriola*. *L. serriola* has hydroxyl (-OH), carboxylic acid (C=O), and alkene (C=C) groups, according to the results of the FTIR analysis. The presence of polyphenolic components in the methanolic extract of *L. serriola* was discovered using HPLC analysis. It is concluded that the methanolic extract of *Lactuca serriola* has hepatoprotective potential, which may be attributed to the presence of polyphenolic components and the antioxidant activities of these compounds [124]. When compared to the group that received only CCl_4_, the ethanol leaf extract of *Launaea taraxacifolia* reduced the acute increase in serum aspartate aminotransferase (AST), alanine aminotransferase (ALT), and alkaline phosphatase (ALP) levels caused by CCl_4_. As a result, it has been hypothesized that the extract has hepatoprotective properties against CCl_4_-induced liver damage through attenuating oxidative stress [125]. The efficacy of an ethanolic extract of lettuce (*Lactuca sativa* L. var. *longifolia*) leaves in protecting the reproductive system of rats against the toxicity produced by carbon tetrachloride (CCl_4_) was investigated. The induction of oxidative stress by CCl_4_ in the rat results in a reduction in the rise in body weight and relative testis weight. It also causes a significant rise in the levels of thiobarbituric acid reactive substances and nitrites in the testicles, as well as a corresponding drop in reduced glutathione and different antioxidant enzymes. CCl_4_ therapy resulted in a drop in the serum levels of testosterone, luteinizing hormone, and follicle-stimulating hormone, as well as a rise in the levels of estradiol and prolactin. The histopathology of CCl_4_-treated rats revealed a partial degeneration of germ and Leydig cells, as well as abnormalities in spermatogenesis, as a result of the treatment. With the use of lettuce extract once a week for ten weeks, the levels of reactive chemicals such as thiobarbituric acid and nitrite are reduced, while the levels of antioxidant enzymes such as catalase, peroxidase, superoxide dismutase, glutathione peroxidase, and glutathione are increased. When CCl4-intoxicated rats were given lettuce extract, the serum levels of testosterone, luteinizing hormone, follicle-stimulating hormone, estradiol, prolactin, histology, body weight, and relative testis weight were all returned to near-normal levels, as did their histology. The findings clearly suggest that therapy with lettuce extract enhances the antioxidant defense system against CCl_4_-induced toxicity. They give evidence that it may have a therapeutic function in treating free radical-mediated diseases in humans [126].

#### 8.1.6. Neuroprotective Activity

The ethyl acetate portion of lettuce prevented the neurotoxic effects of glucose/serum deprivation (GSD) (*Lactuca sativa*). In PC12 cells, lettuce reduced the increased Bax and caspase-3 proteins and decreased Bcl-2 caused by GSD [127]. Lactate dehydrogenase release and 3-(4,5-dimethylthiazol-2-yl)-2,5-diphenyltetrazolium bromide reduction assays were used to assess the neuroprotective effects of the phenolic extract of romaine lettuce and its pure caffeic acid derivatives (isochlorogenic, chlorogenic, chicoric, and caffeic acids) in PC-12 cells. In 100 g of fresh romaine lettuce, total phenolics and total antioxidant capacity averaged 22.7 mg of gallic acid equivalents and 31.0 mg of vitamin C equivalents, respectively. In a dose-dependent manner, the phenolic extract of romaine lettuce rescued PC-12 cells from oxidative damage produced by H_2_O_2_. One of the phenolics in romaine lettuce, isochlorogenic acid, demonstrated more neuroprotection than the other three caffeic acid derivatives discovered in the lettuce. Despite having lower levels of phenolics and antioxidant capacity than other common vegetables, romaine lettuce’s contribution to total antioxidant capacity and anti-neurodegenerative impact in human diets would be greater due to a higher daily per capita intake than other common vegetables [128]. Lettuce glycoside B has a neuroprotective effect on cerebral ischemia-reperfusion damage, which may be due to its capacity to upregulate nerve growth factor and neurotrophin-3 expression in the brain cortex during the late stages of ischemia [129] (Figure 5).

#### 8.1.7. Antioxidant properties

Polyphenol oxidase was extracted from fresh lettuce for the purpose of a research investigation. Both the temperature, which should be kept at 40 °C, and the pH level should be maintained at 7.0. It has been shown that catechin, catechol, chlorogenic acid, caffeic acid, and gallic acid may all be used as substrates by lettuce polyphenol oxidase. The results showed that chlorogenic acid had the highest substrate specificity compared to the other substrates that were tested. Some compounds easily inhibited the activity of lettuce polyphenol oxidase. The enzyme lettuce polyphenol oxidase was subjected to tests against possible inhibitors, including ascorbic acid, cysteine, oxalic acid, and citric acid. Cysteine was the most efficient inhibitor of the three. It also evaluated how much total phenol and total antioxidant activity were present in the sample when these inhibitors were present at room temperature and refrigerator temperature. The total antioxidant activity of lettuce was raised by the addition of ascorbic acid and cysteine, while the addition of citric and oxalic acids had no influence on the level of total antioxidant activity. Ascorbic acid and cysteine worked together to prevent oxidation of the phenolic compounds in lettuce [130]. In a study, the antioxidant activity, both hydrophilic and lipophilic, as well as the total phenolic content of the leaves of three distinct cultivars of *Lactuca sativa* L. (Iceberg, Romaine, and Baby head) were compared and contrasted (stem, inner, medium, and outermost leaves). Romaine had the greatest levels of hydrophilic and lipophilic antioxidant activity, and its phenolic content was also greater than that of Iceberg and Baby head lettuces. Romaine also had the highest phenolic content. While the hydrophilic antioxidant activity exhibits a non-specific distribution, the lipophilic antioxidant activity rose dramatically from the stem to the outermost leaves. This suggests that the lipophilic antioxidant may have a protective function in mature or light-exposed leaves [131]. The influence of pH on the antioxidant activities of combinations of lettuce extract with quercetin, green tea extract, or grape seed extract was investigated. This was undertaken to reduce Fremy’s salt in an aqueous solution using direct electron spin resonance spectroscopy and in l-α-phosphatidylcholine liposome peroxidation assay measured following the formation of conjugated dienes. When employing both of these approaches, the radical-scavenging impact of each and every phenolic antioxidant that was investigated exhibited an increasing trend with rising pH values. When used in combination with lettuce extract, quercetin, green tea extract, and grape seed extract operated in a synergistic manner to prevent the oxidation of peroxidating liposomes, with quercetin demonstrating the greatest benefit. It is owing to an increase in their electron-donating capacity upon deprotonation as well as to their stability in alkaline solutions leading to the polymerization process that the antioxidant activity of the phenols raises with increasing pH. This rise is pH dependent. These polymerization processes of polyphenolic antioxidants might result in enhanced radical scavenging activity as a consequence of the formation of additional oxidizable hydroxyl moieties in the polymeric products of these reactions [131]. Interestingly, both the yield and the antioxidant activity of lettuce are greatly improved by using LED lighting with the spectrum B435/R663 at a ratio of 1.25 ± 0.1 [132]. A higher concentration of certain vitamins, antioxidant enzymes, and polyphenolic compounds is produced in the enhanced plants because of the application of iodine compounds, which improves the antioxidant capacity of lettuce [102].

#### 8.1.8. Other Health Benefits

The mechanism of aging is an accumulation of numerous adverse modifications in the cells and tissues when the ages increase because of free radical/oxidative changes leading to the risks of disease increases. The oxidative damage will happen even in normal conditions. While the damage rates will be high when the aging is due to the poor capacity of antioxidation and repairing [133]. It has been shown that the consumption of antioxidant-rich food, such as a polyphenolic diet, is efficient in decreasing the harmful influence of aging and behavior. Firstly, polyphenols in lettuce are good for alleviating the deleterious effects of aging on the brain and even the nervous system. More specifically, the most important role of food polyphenols in protecting the brain during aging is that these composites can cross the blood–brain barrier (BBB), which strictly controls the flow of metabolites, nutrients, and drugs into the brain. Secondly, Vitamin A belongs to retinoids, which are beneficial for anti-aging skin treatments. Moreover, as another compound, vitamin C is good for your skin that can maintain skin at a young status and accelerate wound healing. Sufficient consumption of vitamin A and vitamin C, which lettuce can be considered a good source, is necessary for developing and maintaining collagen, which provides the structure for the skin.

Regular intake of lettuce can help reduce bone loss. The leafy green vegetable can be considered one of the good natural sources of vitamin K such as romaine lettuce. Some research has found that vitamin K_2_ can help increase bone density and even better reduce the risk of osteoporosis than calcium can. Apart from bone-building and maintaining a skeletal structure in healthy status, vitamin K can also play an important role in blood clotting, treating bruises, and helping bone calcification [134]. Furthermore, we need to metabolize the food we intake properly because it can provide the energy needed for our daily routine. Lettuce consumption can promote the process of metabolism in our body due to the presence of minerals such as iron, magnesium, and potassium. Moreover, the vitamin B complex contained in lettuce is also able to help our metabolism.

### 8.2. Health Risks

Anti-nutrients are the primary factor to cause consumption risks in lettuce. Though the content of these compounds is controlled under a dangerous level in lettuce cultivation, it cannot be ignored; regarding the above-mentioned nitrate, which is perceived as a purely harmful dietary component, its excessive intake can lead to infantile methemoglobinemia, carcinogenesis, and possibly even teratogenesis [135]. Ysart et al. [136] detected an average concentration of 1085 mg/kg (50 to 3209 mg/kg) in several lettuce samples, which was a little over, in the comparison with the European Normative [137]. In the meantime, some metabolites, such as nitrite and nitrogen oxide production, may cause methemoglobinemia and gastric cancer [138]. In addition, the physiological effects alkaloids have on humans are evident [69]. High tropane alkaloid consumption causes fast pulse, paralysis, and death in the worst-case scenario [66]. Negri et al. [139] compared a large number of vegetables, tryptamine content in lettuce is high at 24.5 ± 2.4 µg/g^−1^ of dry weight. Ingestion of large amounts of tryptamine alkaloids causes final death. Moreover, Besharat et al. [140] listed some side effects and toxicity resulting from an overdose of lettuce, including mydriasis and photophobia, dizziness, diaphoresis, auditory hallucination, and cardiovascular and respiratory difficulties caused by dysrhythmia; however, with the herb-use of lettuce, it acts as a spasmolytic and a sedative.

## 9. Future Perspectives

The extraction of bioactive compounds is one of the new trends observed in plants. Some previous studies have shown that lettuce is one of the fascinating and rich sources of antioxidant polyphenols that support human health [141]. Some studies have focused on the extraction of lettuce by various methods. Fresh-cut processing can facilitate the extraction of polyphenols in lettuce tissue, which is a response to cell injury such as leaf-cutting or shredding. As for the procedure used previously, lettuce needs to be homogenized by extractive solution, maintained at a temperature of 4–50 °C for 48 h. Regardless of the time consumed, these procedures cannot provide food-grade polyphenol extracts. Therefore, Chemat et al. [142] reported some new methods using “green” extraction technologies by non-toxic and GRAS solvents with ultrasound assistance that can effectively extract food-grade polyphenol from lettuce waste. The polyphenols and gluconate extraction from lettuce waste can be suitable for functional foods and nutraceuticals.

Another new trend is to make functional flour. Apart from extracting polyphenols from lettuce, it can be used in some other ways. For example, lettuce with high phenol and fiber content is air-dried and ground to add to flour, producing functional baked products. Traditionally, it is common for a bakery to use whole meal flour because of the high antioxidants and fiber contents in bran and aleurone [143]. The same objectives can be reached by flours made from vegetables or their waste, which contains high contents of these compounds. Lettuce flour can be considered an ideal ingredient for bread to increase functionality. Plazzotta et al. [144] verified that lettuce flour greatly improved the phenolic content of bread and increased the antioxidant activity by 200%. Lettuce flour added to bread can gradually reduce the leavening ability of dough and increase the moisture and hardness of bread. However, the utilization of lettuce flour will influence bread processing and sensory aspects of the bread, while bread containing 170 to 575 g/kg of lettuce flour is more acceptable than commercial whole meal bread.

## 10. Conclusions

In conclusion, lettuce has high nutritional value due to its contribution to dietary fiber, several important dietary minerals, various vitamins, and bioactive compounds such as carotenoids, phenolic compounds, chlorophyll, and even sesquiterpene lactones. The bioavailability and metabolism of phytonutrients can better understand related health benefits and the ultimate delivery of these compounds. Especially due to its antioxidant compounds, lettuce can provide some potential health benefits in cardio-protective, anti-cancer, anti-diabetic, and anti-aging. Due to these potentials, both in vitro and in vivo evidence verify the preclinical and clinical application of lettuce extracts, which can be further investigated. Meanwhile, the purified phytochemicals extracted from lettuce may reveal the possibility of producing efficient functional foods. Moreover, further studies can focus on the growing conditions and development strategies to increase the nutritional value of lettuce and widely apply them in other areas.

## Figures and Tables

**Table 1:** Chemical structure of main phytochemicals identified in lettuce.

Phytochemicals	Subgroups	Specific Compounds	Biological Activities	Reference
Phenolic compounds	Phenolic acids	Caffeic acid, Chlorogenic acid, and their derivatives	Antidiabetic, antimicrobial, anti-inflammatory, skin care products, food preservatives	[37,38]
	Flavonoids	Quercetin, Isorhamnetin, Kaempferol, Anthocyanins	Hepatoprotective, antibacterial, anti-inflammatory, anticancer, antiviral	[39,40]
Carotenoids	Carotenes	α-carotene, β-carotene, Lycopene	Cardiovascular protective, anti-cancer, anti-obesity, pigment, antiproliferative	[41,42]
	Xanthophylls	Lutein, Neoxanthin, Lactucaxanthin, Violaxanthin, Zeaxanthin		
Chlorophyll		Chlorophyll A, Chlorophyll B	Antioxidant, anti-cancer, stimulating immune system, pigment, normalize blood pressure	[43,44]

## Data Availability

All data presented herein are constant with the published literature.
